# ZNF281/Zfp281 is a target of miR‐1 and counteracts muscle differentiation

**DOI:** 10.1002/1878-0261.12605

**Published:** 2019-12-24

**Authors:** Sara Nicolai, Marco Pieraccioli, Artem Smirnov, Consuelo Pitolli, Lucia Anemona, Alessandro Mauriello, Eleonora Candi, Margherita Annicchiarico‐Petruzzelli, Yufang Shi, Ying Wang, Gerry Melino, Giuseppe Raschellà

**Affiliations:** ^1^ Medical Research Council Toxicology Unit Department of Pathology University of Cambridge UK; ^2^ Department of Experimental Medicine University of Rome Tor Vergata Italy; ^3^ Istituto Dermopatico dell’Immacolata‐IRCCS Rome Italy; ^4^ CAS Key Laboratory of Tissue Microenvironment and Tumor Shanghai Institute of Nutrition and Health Shanghai Institutes for Biological Sciences Chinese Academy of Sciences University of Chinese Academy of Sciences Shanghai China; ^5^ The First Affiliated Hospital of Soochow University State Key Laboratory of Radiation Medicine and Protection Institutes for Translational Medicine Soochow University Suzhou China; ^6^ Laboratory of Health and Environment ENEA Rome Italy; ^7^ Present address: Ludwig Institute for Cancer Research University of Oxford UK

**Keywords:** miRs, muscle differentiation, soft tissue sarcomas, Zfp281, ZNF281

## Abstract

Defects in achieving a fully differentiated state and aberrant expression of genes and microRNAs (miRs) involved in differentiation are common to virtually all tumor types. Here, we demonstrate that the zinc finger transcription factor ZNF281/Zfp281 is down‐regulated during epithelial, muscle, and granulocytic differentiation *in vitro*. The expression of this gene is absent in terminally differentiated human tissues, in contrast to the elevated expression in proliferating/differentiating ones. Analysis of the 3’UTR of ZNF281/Zfp281 revealed the presence of numerous previously undescribed miR binding sites that were proved to be functional for miR‐mediated post‐transcriptional regulation. Many of these miRs are involved in differentiation pathways of distinct cell lineages. Of interest, ZNF281/Zfp281 is able to inhibit muscle differentiation promoted by miR‐1, of which ZNF281/Zfp281 is a direct target. These data suggest that down‐regulation of ZNF281/Zfp281 during differentiation in various cell types may occur through specific miRs whose expression is tissue‐restricted. In addition, we found that in rhabdomyosarcoma and leiomyosarcoma tumors, the expression of ZNF281/Zfp281 is significantly higher compared with normal counterparts. We extended our analysis to other human soft tissue sarcomas, in which the expression of ZNF281 is associated with a worse prognosis. In summary, we highlight here a new role of ZNF281/Zfp281 in counteracting muscle differentiation; its down‐regulation is at least in part mediated by miR‐1. The elevated expression of ZNF281/Zfp281 in soft tissue sarcomas warrants further analysis for its possible exploitation as a prognostic marker in this class of tumors.

## Graphical abstract

Cellular dedifferentiation is a common feature during malignant transformation. Regulatory and structural proteins and other molecular species such as microRNAs participate in this process. Here, we demonstrate that the transcription factor ZNF281/Zfp281, a target of miR‐1, counteracts muscle differentiation driven by this microRNA. Accordingly, ZNF281/Zfp281 is down‐regulated during muscle differentiation, but its expression increases in human sarcomas.

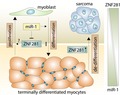

**Abbreviations**
ATRA: *all‐trans*‐retinoic acid
miRs: microRNAs
WB: western blotting

## 1 Introduction

A variable degree of dedifferentiation compared with the normal tissue they originate from is a distinctive feature of tumors (Merrell and Stanger, 2016) and other pathologies (Xie *et al.*, 2018). The gain of progenitor‐like characteristics is associated with cancer progression and spreading to distant locations (metastasis) (Lu and Kang, 2019). Loss of differentiated features during the transformation process can be attributed to a variety of causes such as dysregulation of signaling pathways (Frankson *et al.*, 2017), epigenetic mechanisms (Hu and Shilatifard, 2016), and substantial changes in the transcriptomic profile (Uhlen *et al.*, 2017) of the transformed cells compared with their normal counterparts. Although it has been definitively proved that a high degree of gene expression heterogeneity exists at single‐cell level within a tumor (Tirosh *et al.*, 2016), some genes act as required drivers of more than one differentiation/dedifferentiation process (Saladi *et al.*, 2017; Storm *et al.*, 2016). Among different classes of genes, transcription factors are frequently involved in differentiation for their ability to modulate the expression of tens (or hundreds) of genes simultaneously (Cassandri *et al.*, 2017).

The zinc finger transcription factor ZNF281/Zfp281 (ZNF281 in humans, Zfp281 in mice) acts as a stemness regulator mediating Nanog autorepression during embryonic/fetal life (Fidalgo *et al.*, 2011). An additional role of Zfp281 in maintaining genetic stability of mouse embryos through the repression of L1 retrotransposons has been discovered (Dai *et al.*, 2017). Indeed, the relevance of Zfp281 function during embryonic life is underlined by the lethal phenotype of Zfp281 knockout mice (Fidalgo *et al.*, 2011). Nevertheless, the biological activity of ZNF281/Zfp281 is not limited to embryonic/fetal life; Zfp281 increases cardiac reprogramming of adult mouse fibroblasts by modulating the expression of inflammatory and cardiac genes (Zhou *et al.*, 2017); in normal and transformed cells, ZNF281/Zfp281 down‐regulation promotes osteogenic (Seo *et al.*, 2013) and neuronal differentiation (Pieraccioli *et al.*, 2018), while its expression drives epithelial–mesenchymal transition (EMT) in colon cancer cells (Hahn *et al.*, 2013). A further and somehow unexpected finding highlighted the involvement of ZNF281 in the transcriptional control of DNA damage repair genes (Pieraccioli *et al.*, 2016), as well as in the recruitment of the repair machinery components directly on damage sites (Nicolai *et al.*, 2019).

The multifunctionality of ZNF281/Zfp281 entails that its expression occurs in a variety of tissues of different origins, and consequently, it requires the existence of flexible regulatory mechanisms operating in diverse cellular contexts.

MicroRNAs (miRs) are powerful post‐transcriptional regulators that bind to complementary sequences in their targets provoking their degradation and/or inhibiting their translation (Bartel, 2009). Frequently, miRs act alone or in parallel with transcriptional regulation to fine‐tune the expression of genes involved in complex cellular pathways (Croce and Calin, 2005). Indeed, a wealth of experimental evidence links specific miRs to cellular differentiation as positive (Chen *et al.*, 2006) or negative (Zhang *et al.*, 2014a) regulators of this process. Notably, the same target can be controlled by several miRs depending on their expression in different cellular districts (Lagos‐Quintana *et al.*, 2002). Nevertheless, this adaptable regulation requires the target to have multiple binding sites for different miRs in its 3′UTR. Although miR‐mediated post‐transcriptional inhibition is by far the most documented mechanism of action (Zhu *et al.*, 2017), some miRs can be also involved in other noncanonical processes. For instance, miR‐1, a well‐known driver of muscle differentiation (Chen *et al.*, 2006), is necessary to shuttle Ago2 inside mitochondria of muscle cells and cardiomyocytes where Ago2 promotes the translation of genes involved in ATP synthesis by acting as a mitochondrial translation initiation factor (Zhang *et al.*, 2014b). The latter finding broadens our knowledge on the function of differentiation‐related miRs not only in silencing the expression of genes related to the differentiation process but also in boosting the translation of other genes that help in maintaining the differentiated phenotype. Mutations and/or derangement of miR expression frequently occurs in human neoplasms (Calin *et al.*, 2002) where they can act by inhibiting or promoting neoplastic growth and metastasis (Croce and Calin, 2005; di Gennaro *et al.*, 2018; Hurst *et al.*, 2009; Pekarsky *et al.*, 2018) and by inducing chemoresistance (Si *et al.*, 2018).

Here, we demonstrated that the expression of ZNF281/Zfp281 is repressed in all differentiation processes tested. Its regulation can take place through a post‐transcriptional mechanism operated by several tissue‐restricted miRs. In muscle cells, the prodifferentiation activity of miR‐1 is counteracted by its target Zfp281. The effect of ZNF281/Zfp281 in maintaining an undifferentiated phenotype is exploited in soft tissue sarcomas (Hawkins *et al.*, 2013) where the expression of this gene is elevated compared with normal counterparts and it is associated with a worse prognosis.

## 2 Materials and methods

The study methodologies conformed to the standards set by the Declaration of Helsinki.

### 2.1 Cell lines and treatments

Human HEK293, lung carcinoma H1299, acute promyelocytic leukemia NB4, and murine fibroblast NIH3T3 cell lines were obtained from ATCC (Manassas, VA, USA) and cultured in their suggested medium at 37 °C in 5% CO_2_. Normal human epidermal keratinocytes, neonatal (HEKn; Life Technologies, Thermo Fisher Scientific, Waltham, MA, USA) were cultured in EpiLife medium supplemented with human keratinocyte growth supplements, HKGs (Life Technologies). The cells were kept subconfluent to avoid triggering differentiation. The HEKn cells were seeded on collagen‐coated dishes and were induced to differentiate by adding CaCl_2_ to a final concentration of 1.2 mm to culture medium for 3, 7, and 9 days. Mouse C2C7 cell line, a subclone of C2 myoblasts (Yaffe and Saxel, 1977), was grown to confluency under 5% CO_2_ at 37 °C in Dulbecco’s modified Eagle’s medium (DMEM) supplemented with 20% (v/v) FBS and penicillin–streptomycin (100 U·mL^−1^). Cells were then switched to differentiation medium (DMEM containing 2% horse serum). For granulocytic differentiation, NB4 cells (Lanotte *et al.*, 1991) were kept at 2–5 × 10^5^ and treated with *all‐trans*‐retinoic acid (ATRA) (Sigma, Saint Louis, MO, USA) at concentration of 10 μm for 3, 6, and 9 days. Cell lines utilized were tested for mycoplasma contamination using MycoAlert Mycoplasma Detection Kit (Lonza, Basel, Switzerland) every 3 months.

### 2.2 May–Grünwald–Giemsa staining of NB4 cells

Cytospin preparations of 2 × 10^5^ cells treated or untreated with ATRA were allowed to air‐dry, incubated in pure May–Grünwald solution for 5 min, then in 50% May–Grünwald/water for 10 min, washed in water, and incubated in a 20% Giemsa/water solution for 20 min. The slides were then washed in water, air‐dried, and acquired under a Nikon laser scanning fluorescence confocal microscope (Nikon Eclipse Ti, Nikon, Tokyo, Japan).

### 2.3 Protein analysis and antibodies

Western blot (WB) analysis was carried out as already described (Pieraccioli *et al.*, 2018). Antibodies were ZNF281 (ab101318; Abcam, Cambridge, UK); ΔNP63 (clone 4YA3; Sigma‐Aldrich); c‐Myc (sc‐40; Santa Cruz Biotechnology, Santa Cruz Biotechnology, Inc., Dallas, TX, USA); K10 (Covance, Princeton, NJ, USA); MyoG (sc‐1273; Santa Cruz); anti‐Myosin (M8421; Sigma‐Aldrich); β‐actin (AC‐15 a5441; Sigma); anti‐β tubulin (sc‐9104; Santa Cruz Biotechnology, Dallas, Texas USA); anti‐mouse‐HRP‐conjugated (Bio‐Rad, Hercules, CA, USA; Cat. No.: 170‐5047); and anti‐rabbit‐HRP‐conjugated (Bio‐Rad; Cat. No.: 170‐6515).

### 2.4 Cell transfection, RNA extraction, and real‐time qPCR analyses

Pre‐miRNAs**,** anti‐miRNA‐1, and siRNAs indicated in Table S3 were used for transfection of H1299 or C2C7 cells according to the manufacturer’s instructions using Lipofectamine RNAiMAX Transfection Reagent (Invitrogen, Carlsbad, CA, USA). mRNA was extracted with the RNeasy Mini Kit 50 (Qiagen, Hilden, Germany) according to the standard procedures. Reverse transcription and qPCR were carried out as previously described (Pieraccioli *et al.*, 2016). hβ‐actin and mGAPDH were used as internal control. Oligonucleotides used in this study are listed in Table S2. The miRNA extraction, reverse transcription, and qPCR were carried out as previously described (Pieraccioli *et al.*, 2016). TaqMan MicroRNA Assay Kits for miR‐1 (#002222), using sno202 (#001232) as an endogenous control [Applied Biosystems (Waltham, MA, USA), Life Technologies], were used in this study.

### 2.5 Cloning

3′UTR of human ZNF281 or murine Zfp281 was amplified using the primers listed in Table S2 and cloned in pGL3‐control vector (Promega, Madison, WI, USA) by standard cloning procedures. Murine Zfp281 coding sequence deprived of 3′ UTR was amplified from cDNA obtained from proliferating C2C7 myoblast and cloned into pLenti‐CMV‐GFP‐2A‐Puro (ABM Inc., Vancouver, Canada) by using the primers listed in Table S2. Deletion mutants for miRNA binding sites were generated by amplification of the PGL3‐control‐ZNF281‐3′UTR plasmid used as a template using the primers listed in Table S2. The sequence of all plasmids generated for this paper was checked by dideoxy‐sequencing.

### 2.6 Dual‐Luciferase reporter assay

Cells were transfected with wild‐type and mutant reporters containing the wt and the mutated 3′UTR of human ZNF281, or wild‐type reporter containing the 3′UTR of mouse Zfp281. Cells were cotransfected with the pre‐miRNAs indicated in Table S3 or negative control using Lipofectamine 2000 (Invitrogen) according to the manufacturer’s suggestions. Luciferase assays were carried out using the Dual‐Luciferase Reporter Assay (Promega). Each experimental point was analyzed in triplicate in three independent experiments. Statistical significance was calculated by unpaired two‐tailed Student’s *t*‐test.

### 2.7 Immunohistochemical staining

For optimization of conditions of immunohistochemistry, skin biopsy formalin‐fixed paraffin‐embedded sections were dewaxed and rehydrated, and blocked for 20 min in 0.03% hydrogen peroxide solution in methanol; then, heat‐mediated antigen retrieval (HIER) was performed by boiling the samples in either 0.01 m citrate buffer pH 6.0 or 1 mm EDTA/0.05%/Tween‐20 pH 9.0 for 30 min in microwave. Then, the slides were incubated with anti‐ZNF281 antibodies purchased from either Sigma (1 : 50, Cat. No. HPA051228; Sigma) or Abcam (1 : 50, Cat. No. Ab154093; Abcam) for 2 h at RT. For further immunohistochemical staining of normal and tumor tissues, HIER was performed by boiling the samples in 0.01 m citrate buffer pH 6.0 for 1 h in microwave; then, samples were blocked in 5% normal goat serum in PBS and stained with anti‐ZNF281 antibody Ab154093 (Abcam) at 1 : 50 dilution overnight at +4 °C. The signal was detected using UltraTek HRP Anti‐Polyvalent DAB Staining System (ScyTek, Logan, UT, USA); then, the slides were counterstained with hematoxylin, dehydrated, and mounted. Tissue microarray (TMA) of soft tissue tumors was purchased from US Biomax (Cat. No. SO751a; US Biomax, Rockville, MD, USA). Other samples of normal skeletal and smooth muscle (additional 10 cases), skin, tonsil, colon, adipose tissue, and liposarcoma were utilized with the approval of the institutional review board of University Hospital ‘Policlinico Tor Vergata’ (Protocol No. 129/18, Rome, Italy). Prior patient consent was informed and written. All the slides were scanned using 40× objective in the Ventana iCoreo scanner (Ventana, Oro Valley, AZ, USA).

### 2.8 Histological scoring of the samples

Samples were scored in a blinded manner by a pathologist using a semiquantitative method. Cases were analyzed for staining intensity, which was scored as 0 (not detected), 1+ (weak), 2+ (intermediate), and 3+ (strong). For each case, the histological *H*‐score (0–300) (Budwit‐Novotny *et al.*, 1986) was calculated by multiplying the percentage of positive cells (0–100%) by the staining intensity (0–3).

### 2.9 Bioinformatic analysis

For the analysis of RNA‐seq carried out in myoblasts, the signal tracks and transcript quantifications were downloaded from ENCODE data portal (http://www.encodeproject.org). Signal tracks were visualized using Integrated Genome Browser (bioviz.org). The following experiments were analyzed: differentiation of human primary myoblasts (ENCSR444WHQ and ENCSR828TEI) and differentiation of murine‐immortalized myoblasts C2C12 (ENCSR000AHY and ENCSR000AIA). For gene expression analyses, normalized values of ZNF281 expression were obtained from NCBI Gene Expression Omnibus (http://www.ncbi.nlm.nih.gov/geo/query/acc.cgi?acc=GSE108022, http://www.ncbi.nlm.nih.gov/geo/query/acc.cgi?acc=GSE62544, http://www.ncbi.nlm.nih.gov/geo/query/acc.cgi?acc=GSE17674, http://www.ncbi.nlm.nih.gov/geo/query/acc.cgi?acc=GSE114621, http://www.ncbi.nlm.nih.gov/geo/query/acc.cgi?acc=GSE6011, http://www.ncbi.nlm.nih.gov/geo/query/acc.cgi?acc=GSE38417, and http://www.ncbi.nlm.nih.gov/geo/query/acc.cgi?acc=GSE21122). For survival analyses, clinical and gene expression data were obtained from TCGA (TCGA_SARC) or NCBI GEO (http://www.ncbi.nlm.nih.gov/geo/query/acc.cgi?acc=GSE17674).

### 2.10 Statistical analysis

All statistical analyses were performed using graphpad prism 8.0 software (GraphPad Software, San Diego, CA, USA). For the analysis of gene array data and protein level of ZNF281 from the TMA experiment, the significance level (P) was calculated using the Mann–Whitney test. The Kaplan–Meier curves were generated in graphpad prism 8.0 software, and the significance level was calculated using the log‐rank Mantel–Cox test. Values of *P* < 0.05 were considered significant. Violin plots were generated in R using ggplot2 package.

## 3 Results

### 3.1 Post‐transcriptional regulation of ZNF281 is mediated by miRs

**Figure 1:**
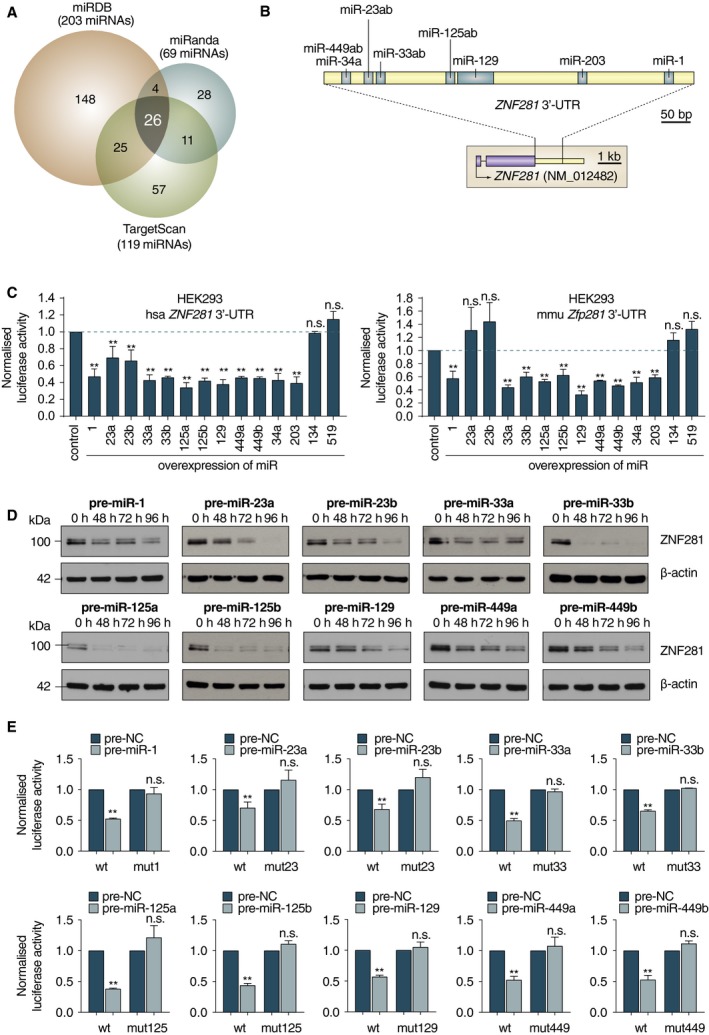
(A) Venn diagram showing predicted miR binding sites on human ZNF281 3’UTR with miRDB, miRanda, and Target Scan online databases. (B) Schematic representation of the human ZNF281 3′UTR. Blue boxes indicate the binding sites of differentiation‐related miRs selected for further analysis. (C) Luciferase assays carried out in HEK293 cells cotransfecting the indicated miRs with reporter vectors for human ZNF281 3’UTR (left) or mouse Zfp281 3′UTR (right). Graphs present means ± SD; **P < 0.01 (two‐tailed Student’s *t*‐test). (D) H1299 cells were transfected with the indicated miRs and collected at different time points. WB analysis demonstrates ZNF281 decrease; β‐actin was used as a loading control. (E) Luciferase assays performed as in (D) with mutated reporter vectors for human ZNF281 3’UTR. Graphs present means ± SD; **P < 0.01 (two‐tailed Student’s *t*‐test).

Since ZNF281/Zfp281 has been detected in many normal tissues during embryonic and adult life (Fidalgo *et al.*, 2011; Pieraccioli *et al.*, 2018; Zhou *et al.*, 2017), we looked for a flexible regulatory mechanism that can effectively regulate its expression in different cellular contexts. We analyzed the 3′UTR of the human ZNF281 gene looking for potential miR binding sites with three commonly used bioinformatic programs (miRDB, miRanda, and Target Scan). Together with the previously described sites for miR‐203 (Viticchie *et al.*, 2011) and miR‐34a (Hahn *et al.*, 2013; Pieraccioli *et al.*, 2018), we found 24 additional sites recognized by all the three programs utilized that were not previously described (Fig. 1A and Table S1). We noticed that many of these miRs were involved in cellular differentiation (Table 1), suggesting the existence of a general regulatory mechanism to down‐regulate ZNF281 during various differentiation processes. To test this hypothesis, we selected 12 of these differentiation‐related miRs (Fig. 1B) and we carried out functional assays by transfecting reporter vectors containing the human 3′UTR of the ZNF281 gene and individual pre‐miRs. All miRs analyzed (miR‐1, miR‐23a, miR‐23b, miR‐33a, miR‐33b, miR‐125a, miR‐125b, miR‐129, miR‐449a, miR‐449b, miR‐34a, and miR‐203) were able to significantly down‐regulate luciferase activity from the human ZNF281 3′UTR (Fig. 1C, left). The same miRs excluding miR‐23a and miR‐23b could also down‐regulate the murine 3′UTR of Zfp281 (Fig. 1C, right). In this analysis, miR‐134 and miR‐519, for which no binding sites were found in the 3′UTRs of ZNF281/Zfp281, were kept as negative controls (Fig. 1C). A comparison between the human and murine 3’UTRs revealed that the seed sequence for miR‐23a/b that is present in humans is not conserved in mice in contrast to the seed sequences of the other regulatory miRs tested (Fig. S1A,B). In addition, to rule out the possibility that miR‐23a/b could regulate murine Zfp281 at the translational level, we transfected the murine NIH3T3 cells with pre‐miR‐23a and pre‐miR‐23b and analyzed the level of Zfp281 protein by western blotting (WB). The amount of Zfp281 protein did not significantly change in miR‐23a/b‐transfected cells compared with controls (Fig. S1C), confirming that miR23a/b are unable to affect the expression of Zfp281 in mice.

**Table 1:** Differentiation‐related miRs that control ZNF281/Zfp281 expression.

miR	Differentiation pathways	References
miR‐1/miR206	Muscle	Chen *et al. *(2006)
miR‐23ab	Neural, EMT	Kawasaki and Taira (2003); Yang *et al. *(2017)
miR‐33ab	EMT	Qu *et al. *(2015); Yang *et al. *(2015)
miR34abc	Brain, EMT, ciliogenesis, spermatogenesis	Agostini *et al. *(2011); Bao *et al. *(2012); Bouhallier *et al. *(2010); Hahn *et al. *(2013); Walentek *et al. *(2016)
miR‐125ab	Neural, EMT	Boissart *et al. *(2012); Cowden Dahl *et al. *(2009); Ottaviani *et al. *(2018)
miR‐129	EMT	Liu *et al. *(2014)
miR‐203	Epithelial, EMT	Diao *et al. *(2014); Viticchie *et al. *(2012); Wellner *et al. *(2009)
miR‐382	Granulocyte	Zini *et al. *(2016)
miR‐449ab	Brain, ciliogenesis spermatogenesis	Li *et al. *(2018); Wu *et al. *(2014)
miR‐495	Muscle, mesendoderm	Xie *et al. *(2019); Yang *et al. *(2014)

The miRs that were able to down‐regulate the expression ZNF281/Zfp281 (Fig. 1C) were further tested for their ability to inhibit the expression of ZNF281 by transfecting their pre‐miR forms in H1299 cells and measuring the level of ZNF281 protein at different time points by WB analysis (Fig. 1D). To understand whether the post‐transcriptional regulation of ZNF281 by these differentiation‐related miRs was dependent on their binding to the corresponding site in the 3'UTR of ZNF281, we performed functional assays in which we cotransfected the 3'UTR mutant forms of ZNF281 where the potential sites for each of the analyzed miRs were deleted by site‐directed mutagenesis (Fig. S1D), together with each of the analyzed miRs. Our analysis indicates that ZNF281 regulation by differentiation‐related miRs occurs through the binding to their respective sites in the 3′UTR of this gene (Fig. 1E). Together, these data highlight a post‐transcriptional regulation of ZNF281/Zfp281 mediated by several miRs whose expression was associated with differentiation.

### 3.2 The expression of ZNF281/Zfp281 decreases during differentiation of tissues and cells of different lineages

**Figure 2:**
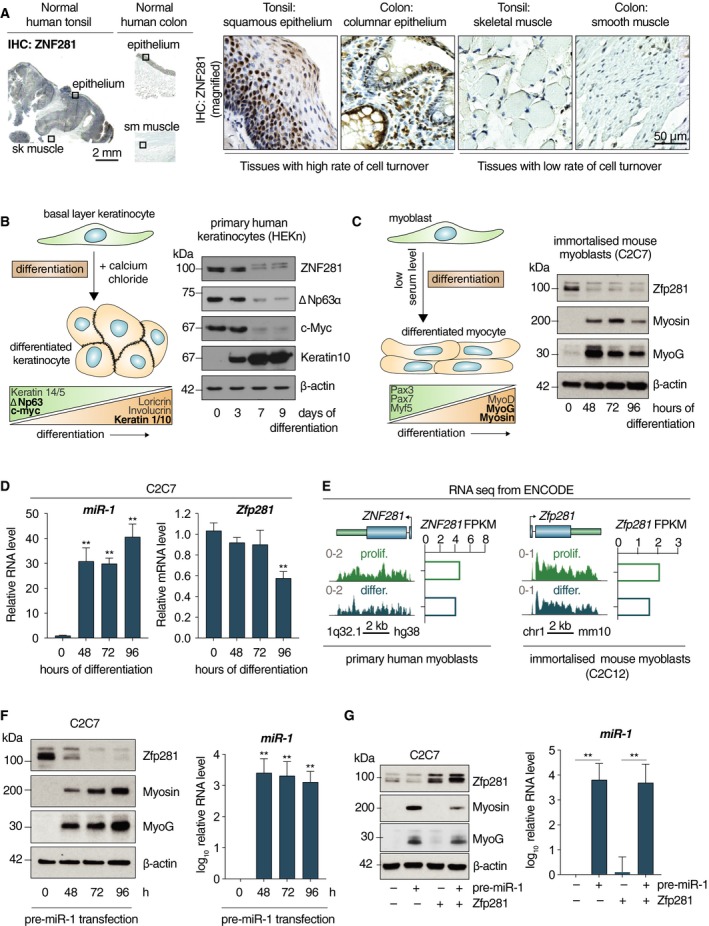
(A) Immunostaining of ZNF281 on either normal tonsil with adjacent skeletal muscle or normal colon with adjacent smooth muscle. Scale bars are 2 mm (on the left) and 50 µm (on the right). (B) Schematic representation of primary human keratinocytes *in vitro* differentiation (left). Markers of proliferation and differentiation are shown on the bottom. The proteins analyzed for WB (right) are in black; β‐actin was used as a loading control. (C) Schematic representation of mouse‐immortalized myoblasts *in vitro* differentiation (left). Markers of proliferation and differentiation are shown on the bottom. The proteins analyzed for WB (right) are in black; β‐actin was used as a loading control. (D) C2C7 cells were induced to differentiate for the indicated time points. qPCR analysis show miR‐1 (left) and Zfp281 (right) levels. Graphs present means ± SD; **P < 0.01 (two‐tailed Student’s *t*‐test). (E) mRNA expression of human *ZNF281* and murine *Zfp281* analyzed by RNA‐seq from ENCODE carried out in the human primary myoblasts (left) or murine C2C12‐immortalized myoblasts (right) in conditions of both proliferation and differentiation. Graphs on the right show the quantification of transcript abundance as fragments per kilobase million. (F) C2C7 cells were transfected with pre‐miR‐1 and harvested at the indicated time points. WB analysis showing the expression of the indicated proteins; β‐actin was used as a loading control (left). qPCR analysis proving miR‐1 overexpression (right). Graphs present means ± SD; **P < 0.01 (two‐tailed Student’s *t*‐test). (G) WB analysis of C2C7 cells transfected as indicated; β‐actin was used as a loading control (left). qPCR analysis demonstrating miR‐1 overexpression (right). Graphs present means ± SD; **P < 0.01 (two‐tailed Student’s *t*‐test).

The expression of ZNF281 was evaluated in different human tissues using a specific antibody (see antibodies tests and procedures in Fig. S2A). ZNF281 is clearly detected in the nuclei of cells in the basal layer of normal squamous epithelium (tonsil) and in the proliferating layer of the columnar epithelium (colon) (Fig. 2A). On the contrary, it was undetectable in post‐mitotic, terminally differentiated tissues such as skeletal and smooth muscle (Fig. 2A). In addition, human keratinocyte precursors (HEKn) were allowed to differentiate *in vitro* toward an epithelial phenotype in medium containing CaCl_2_ (Fig. 2B, left). The expression of proliferation (c‐Myc and ΔNp63) and differentiation (Keratin 10) markers was evaluated by WB analysis during the differentiation process. As expected, the expression of ΔNp63 and c‐Myc decreased after 7 days in culture, while Keratin 10 (K10) sharply increased from day 3 up to day 9 (Fig. 2B, right). Of interest, the expression of ZNF281 rapidly declined from day 3 to 9 (Fig. 2B, right). To evaluate whether the decrease in ZNF281/Zfp281 was a phenomenon occurring also during other differentiation pathways, we tested the immortalized murine myoblasts, C2C7 cells (Yaffe and Saxel, 1977), which recapitulate muscle differentiation *in vitro* upon lowering serum level in the medium (Fig. 2C, left). In this case, the expression of Zfp281 protein drastically dropped after 48 h in differentiation medium (Fig. 2C, right). In parallel, the muscle markers Myosin and MyoG increased (Fig. 2C, right). Furthermore, we tested the granulocytic differentiation of the human promyelocytic leukemia cells NB4 (Lanotte *et al.*, 1991) by treatment with ATRA (Fig. S2B). WB analysis demonstrated a strong reduction of ZNF281 signal upon RA treatment (Fig. S2C), while only a partial but significant decline in its transcript was detected (Fig. S2D). In the same time frame, c‐Myc and MPO decreased and the differentiation markers CD11B, CD11C, and CD14 increased (Fig. S2D). Altogether, these data indicate that ZNF281/Zfp281 decrease is associated with the acquisition of a differentiated phenotype in different cellular lineages.

### 3.3 Functional relationship between miR‐1 and Zfp281 during differentiation of murine myoblasts

miR‐1 expression is tissue‐restricted prevalently in muscle where it was demonstrated to function as a powerful driver of myogenic differentiation through different mechanisms (Chen *et al.*, 2006; Zhang *et al.*, 2014b). To further test the role of miR‐1 in muscle differentiation, we transfected C2C7 cells with anti‐miR‐1 and cultured them in differentiation medium for 6 and 24 h. As expected, anti‐miR‐1‐transfected cells expressed a lower amount of the differentiation marker MyoG compared with controls by WB analysis (Fig. S2E). Next, since miR‐1 is able to inhibit the expression of ZNF281/Zfp281, we monitored the expression of miR‐1 during C2C7 differentiation. We detected a sharp increase (around 30 folds) in miR‐1 expression already after 48 h in differentiation medium with a further increase at 96 h (Fig. 2D). Notably, while the expression of Zfp281 protein abruptly dropped at 48 h (Fig. 2C), its mRNA levels significantly decreased only at late differentiation time (96 h) (Fig. 2D). The slow decline in ZNF281/Zfp281 mRNA during muscle differentiation was confirmed by RNA‐seq analysis of primary human myoblasts and immortalized mouse myoblasts (Fig. 2E). Indeed, we could appreciate only a partial reduction of the levels of ZNF281/Zfp281 transcripts in differentiated compared with undifferentiated cells (Fig. 2E). Thus, the slow decrease in Zfp281 mRNA together with the fast reduction of the protein levels is consistent with a miR‐mediated post‐transcriptional regulation. To better understand the functional relationship between Zfp281 and miR‐1, we induced myogenic differentiation in C2C7 cells by transfecting pre‐miR‐1 and evaluating the expression of muscle differentiation markers Myosin and MyoG and the level of Zfp281 protein by WB analysis (Fig. 2F). We could detect a sharp up‐regulation of Myosin and MyoG paralleled by a decrease in Zfp281 (Fig. 2F). As expected, the levels of miR‐1 dramatically increased after pre‐miR‐1 transfection and remained high during the time frame of the differentiation experiment (Fig. 2F). Next, we sought to rescue the prodifferentiation effect of miR‐1 on C2C7 cells by sequentially transfecting miR‐1 and a vector containing only the coding sequence of the Zfp281 gene (without the 3′UTR) (Fig. 2G). Cells transfected with miR‐1 and the empty vector underwent differentiation as expected (Fig. 2G). Of note, cotransfection of the Zfp281 expression vector caused a marked reduction in the expression of the late differentiation marker Myosin and a modest down‐regulation of the early marker MyoG compared with the cotransfection with empty vector (Fig. 2G). Thus, miR‐1‐mediated muscle differentiation occurs, at least in part, through a reduction of Zfp281 protein that, in turn, is able to counteract the prodifferentiation effect of miR‐1. To understand whether the inhibition of Zfp281 was able *per se* to accelerate the differentiation process, we transfected C2C7 cells with siRNA directed against Zfp281. This experiment suggests that although the reduction of Zfp281 is a requisite for muscle differentiation, its sole inhibition does not increase the kinetics of the process as evaluated by the lack of induction of the MyoG marker (Fig. S2F).

### 3.4 The expression of ZNF281 is predictive of prognosis in soft tissue sarcomas

**Figure 3:**
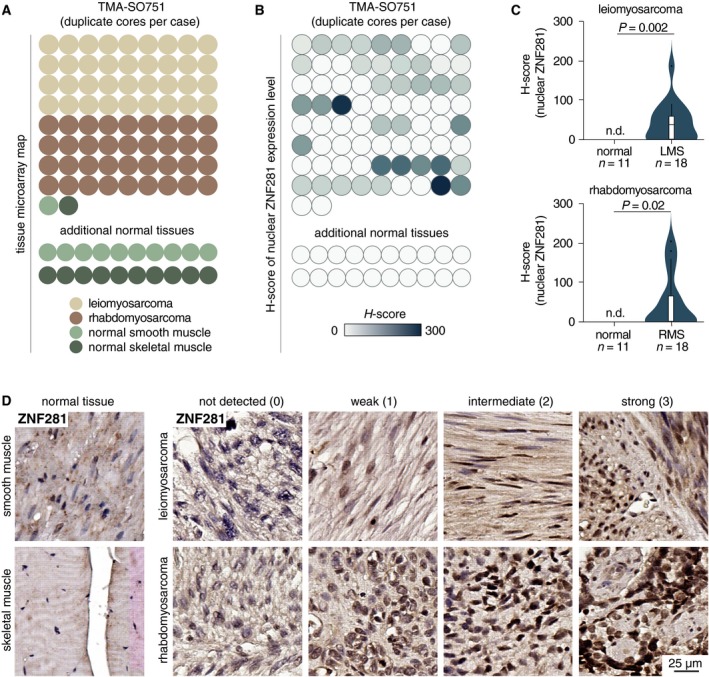
(A) The structure of TMA of soft tissue cancer (SO751a; US Biomax) showing the distribution of samples. Twenty additional normal smooth and skeletal muscle samples were collected from University Hospital ‘Policlinico Tor Vergata’ and used as further controls. (B) Heatmap showing the distribution of *H*‐scores of nuclear ZNF281 expression in the TMA from (A). (C) Violin plots comparing *H*‐scores of nuclear ZNF281 level between normal tissue and soft tissue cancer samples from (B). (D) Representative immunostaining of ZNF281 on samples of normal and tumor samples from TMA from (A). Scale bar is 25 µm.

Our data indicate that the expression of ZNF281 decreases during muscle differentiation, while the ectopic expression of this gene counteracts the miR‐1‐driven differentiation of C2C7 myoblasts. Thus, we analyzed samples of normal muscle tissue (*N* = 11 for smooth muscle and *N* = 11 for skeletal muscle) and a TMA containing leiomyosarcomas (Serrano and George, 2013) (*N* = 18) and rhabdomyosarcomas (Skapek *et al.*, 2019) (*N* = 18) (Fig. 3A). We found that ZNF281 is expressed at variable levels in both tumor types (Fig. 3B). Conversely, ZNF281 was undetectable in all normal skeletal and smooth muscle specimens analyzed (Fig. 3B). ZNF281 levels expressed as *H*‐score (Budwit‐Novotny *et al.*, 1986) are higher in leiomyosarcoma and rhabdomyosarcoma compared with their normal counterparts (*P* = 0.002 and *P* = 0.02, respectively; Fig. 3C). A complete description of the histopathological features and the *H*‐score of ZNF281 expression for each tumor analyzed is summarized in Table S4. Representative immunostaining of ZNF281 in normal and tumor samples is presented in Fig. 3D.

**Figure 4:**
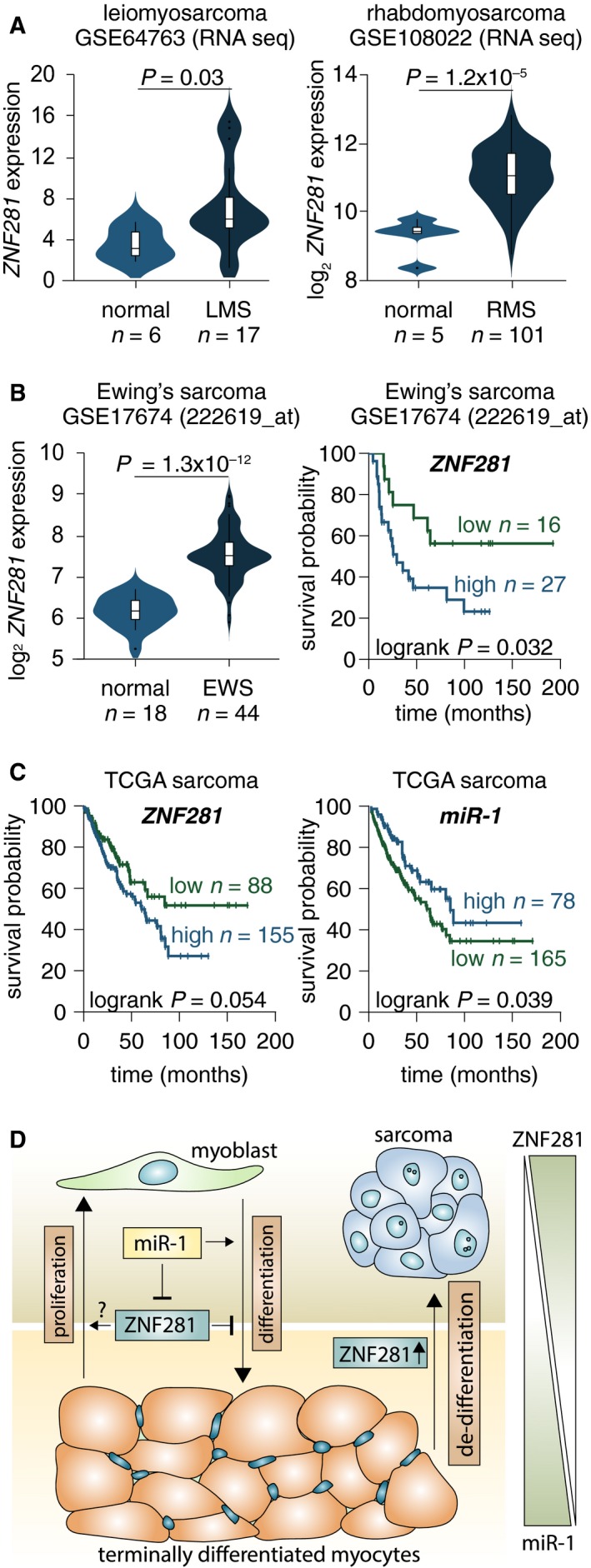
(A) Violin plots comparing *ZNF281* expression between normal tissue and soft tissue cancer samples from the studies http://www.ncbi.nlm.nih.gov/geo/query/acc.cgi?acc=GSE64763 (leiomyosarcoma) and http://www.ncbi.nlm.nih.gov/geo/query/acc.cgi?acc=GSE108022 (rhabdomyosarcoma). (B) Violin plots comparing *ZNF281* expression between normal tissue and Ewing’s sarcoma samples from the study http://www.ncbi.nlm.nih.gov/geo/query/acc.cgi?acc=GSE17674. On the right panel, the Kaplan–Meier survival analysis based on expression levels of *ZNF281* in the Ewing’s sarcoma samples from http://www.ncbi.nlm.nih.gov/geo/query/acc.cgi?acc=GSE17674. (C) The Kaplan–Meier survival analysis based on expression levels of either *ZNF281* or *miR‐1* in the sarcoma samples from TCGA. (D) Scheme showing the expression levels of and the link between ZNF281 and miR‐1 during muscle differentiation and sarcoma progression.

In agreement with the results obtained with TMA, the analysis of publicly available datasets of soft tissue sarcomas confirmed a significantly higher expression of ZNF281 in leiomyosarcoma, rhabdomyosarcoma, dedifferentiated liposarcoma, and myxoid/round cell liposarcoma (Hawkins *et al.*, 2013) patients compared with normal counterparts (Figs 4A and S3A,B). Furthermore, a tendency to higher expression of ZNF281 was also detected in a panel of 46 sarcoma cell lines compared with nontransformed cell lines from soft tissues (Fig. S3C). The Kaplan–Meier survival probability analysis in Ewing’s sarcoma performed by subdividing patients in high‐ and low‐expressing ZNF281 highlighted a significantly worse prognosis in high expressors (Fig. 4B, log‐rank *P* = 0.032). Interestingly, in the TCGA sarcoma dataset, the Kaplan–Meier survival probability analysis indicated that high miR‐1 expression identifies a subset of patients with better outcome compared with the low expressors (Fig. 4C, log‐rank *P* = 0.039). An opposite trend was detected for ZNF281 for which high expression tends to be predictive of worse outcome (Fig. 4C, log‐rank *P* = 0.054). The latter result is consistent with the post‐transcriptional control exerted by miR‐1 on ZNF281 expression that we have demonstrated *in vitro*. Altogether, TMA and bioinformatic analyses suggest that the expression of ZNF281 is elevated in a wide range of soft tissue sarcomas.

## 4 Discussion

Here, we demonstrate that down‐regulation of ZNF281/Zfp281 expression is a common feature of several differentiation pathways (epithelial, myogenic, and granulocytic). These data suggest that the decrease in ZNF281/Zfp281 expression is a general requisite for the completion of differentiation in somatic cells and raise the question of how the regulation of this factor occurs in different cellular contexts.

The analysis of the ZNF281/Zfp281 3’UTR revealed the presence of numerous miR binding sites that suggested a miR‐mediated post‐transcriptional control exerted on this gene. We demonstrated that a large number of these miRs were indeed able to regulate the expression of ZNF281/Zfp281. Since many of these miRs have tissue‐restricted expression, it is conceivable that the miR‐mediated post‐transcriptional control of ZNF281/Zfp281 during various types of cellular differentiation occurs through miRs primarily expressed in a specific cellular context.

To address this issue, we focused our study on muscle differentiation and on miR‐1, whose expression is tissue‐restricted in skeletal muscle and heart and which is itself a driver of myogenic differentiation acting through canonical and noncanonical mechanisms (Chen *et al.*, 2006; Zhang *et al.*, 2014b). Our data demonstrate that ZNF281/Zfp281 is a new target of miR‐1 in muscle cells. The functional relationship between miR‐1 and ZNF281/Zfp281 is highlighted by the inhibitory effect that ectopic expression of this gene has on miR‐1‐driven muscle differentiation of C2C7 myoblasts. The latter finding also implies that ZNF281/Zfp281 is itself a negative modulator of myogenic differentiation although its sole inhibition is unable to accelerate the process. Thus, our data indicate that miR‐1 exerts a post‐transcriptional control on the expression of ZNF281/Zfp281 (through direct binding to the 3′UTR of ZNF281/Zfp281) to ensure the down‐regulation of this gene that is required for the achievement of a differentiated state. Indeed, the expression of ZNF281/Zfp281 is no longer required in fully differentiated myotubes as demonstrated by our immune‐histological analysis of skeletal and smooth muscle tissues. A scheme of the roles of ZNF281/Zfp281 and miR‐1 in muscle differentiation is presented in Fig. 4D. Complex events such as cell differentiation require adjustments of gene expression that must be time‐, point‐, and cell‐lineage‐specific and involve numerous players (Strober *et al.*, 2019). Our data do not rule out that the regulation of ZNF281/Zfp281 can also occur through other mechanisms; however, the miR‐1‐mediated gene regulation during myogenic differentiation seems to be appropriate for controlling more than one target by only one effector.

Contrary to what happens in skeletal and smooth muscle tissues, the expression of ZNF281/Zfp281 is significantly elevated in rhabdomyosarcoma (Shern *et al.*, 2014) and leiomyosarcoma (Hernando *et al.*, 2007) tumors. This observation suggests that the expression of ZNF281 can be considered a marker of proliferative/transformed state related to the dedifferentiation process common to virtually any type of tumor (Merrell and Stanger, 2016). Our analysis also reveals that elevated expression of ZNF281 compared to normal counterparts is a feature of several other soft tissue sarcomas (van der Graaf *et al.*, 2017). In line with this, high expression of ZNF281 is associated with a significantly worse prognosis in Ewing’s sarcoma (Balamuth and Womer, 2010).

## 5 Conclusion

In summary, our study highlights a common behavior of ZNF281/Zfp281 during several differentiation pathways in which the expression of this gene is invariably down‐regulated. Modulation of the expression of this gene is, at least in part, dependent on post‐transcriptional control of different miRs that are frequently tissue‐restricted. Accordingly, in muscle differentiation, a functional inverse relationship exists between Zfp281 and muscle‐specific miR‐1. The elevated expression of ZNF281 in a vast range of soft tissue sarcomas warrants further investigation of the prognostic potential of this gene within this class of deadly tumors.

## Conflict of interest

The authors declare no conflict of interest.

## Author contributions

SN, MP, AS, GM, and GR designed the experiments. GR, EC, and GM supervised the project. SN MP, AS, and CP performed the biochemical experiments. LA and AM performed the histopathological evaluation. AS, SN, and MP performed the bioinformatic analysis. SN, MP, AS, GR, EC, MAP, YW, YS, and GM analyzed the data. SN, GR, and GM wrote the manuscript, and all the authors commented and edited the manuscript.

## Supporting information


**Fig. S1**. (A) Sequence alignment of the indicated miRs on human ZNF281 3’UTR. (B) Schematic representation of the human ZNF281 3’UTR and murine Zfp281 3’UTR indicating the binding sites of differentiation‐related miRs selected for further analysis. (C) Murine NIH3T3 cells were transfected with the indicated miRs and collected at different time points. WB analysis demonstrates that Zfp281 is not under control of miR‐23a/b; β‐actin was used as a loading control. The asterisk indicates non‐specific band. (D) Schematic representation of ZNF281 3’UTR different mutants. Red boxes indicate deletions of the relative binding sites along ZNF281 3’UTR.

**Fig. S2**. (A) Immunostaining of ZNF281 on normal human skin. Two different antibodies and three different conditions of heat‐induced epitope retrieval (no HIER, HIER pH9 EDTA, and HIER pH6 Citrate) were used to determine the optimal condition for immunostaining. (B) Representative images of NB4 cells treated with DMSO or ATRA 10 µM for 9 days. (C) WB analysis of NB4 cells induced to differentiate for the indicated times; ‐actin was used as a loading control. (D) qPCR analysis of samples in (C). (E) WB analysis of C2C7 cells treated with the indicated RNA oligonucleotides for 24 h and then shifted in differentiation medium for either 6 or 24 h; ‐tubulin was used as a loading control. (F) WB analysis of C2C7 cells transfected with the indicated siRNAs for 48h; ‐tubulin was used as a loading control.

**Fig. S3**. (A) Violin plots comparing *ZNF281* expression between normal adipose tissue and different types of liposarcoma from the study GSE21122. (B) Immunostaining of ZNF281 on either normal human adipose tissue of breast or de‐differentiated liposarcoma. Infiltrating lymphocytes were used as internal positive control for ZNF281 immunostaining of breast, meanwhile smooth muscle adjacent to tumor was used as internal negative control for specificity of ZNF281 immunostaining of liposarcoma. (C) A heatmap showing the relative mRNA expression of *ZNF281* in either 5 normal cell lines of soft tissues or 46 cell lines of soft tissue cancer.

**Fig. S4**. Uncropped western blots related to Figs 1D and 2B,C.

**Fig. S5**. Uncropped western blots related to Fig. 2F,G and to Fig. S2C.

**Table S1** **.** Bioinformatic analysis of miRNA sites in human ZNF281 3'‐UTR.

**Table S2** **.** Oligonucleotides used in the study.

**Table S3** **.** Pre‐miR, Anti‐miR and siRNAs used for transfection.

**Table S4** **.** Tissue microarray TMA‐SO751a from US Biomax (leiomyosarcoma/rhabdomyosarcoma duplicate cores per case).
