# Supplementary figures and images for: ZNF281/Zfp281 is a target of miR‐1 and counteracts muscle differentiation
Source: Mol Oncol. 2019 Dec 24;14(2):294–308. doi: 10.1002/1878-0261.12605 (PMC6998661; doi:10.1002/1878-0261.12605)

# Supplementary Figure S1 related to Figure 1

**A**

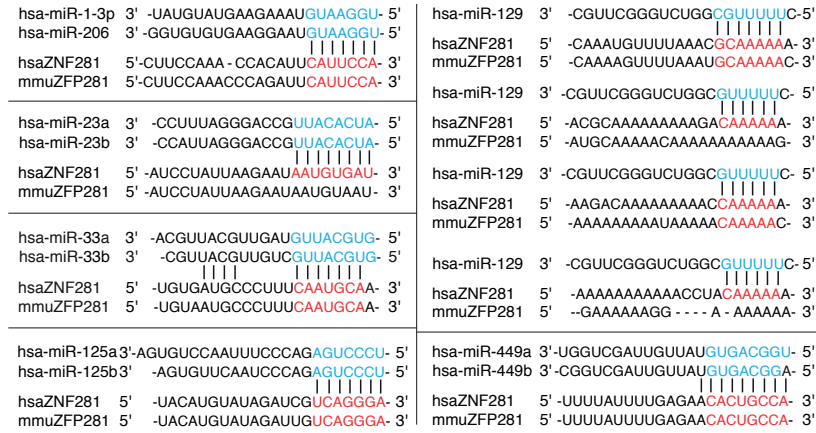

**B**

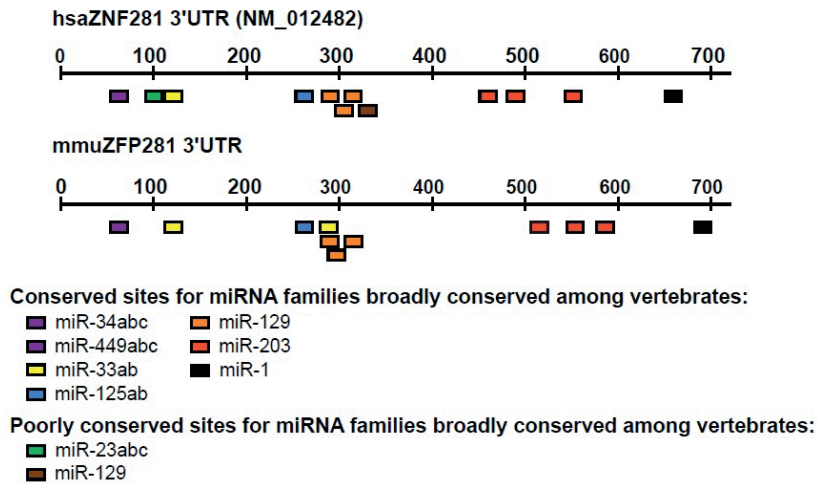

**C**

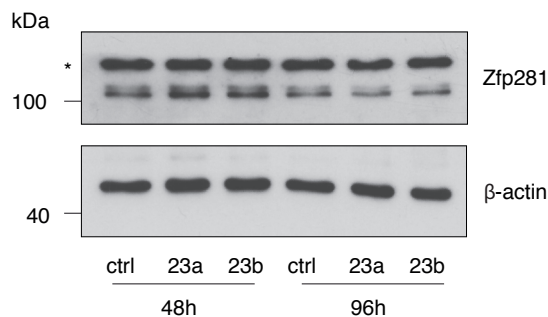

**D**

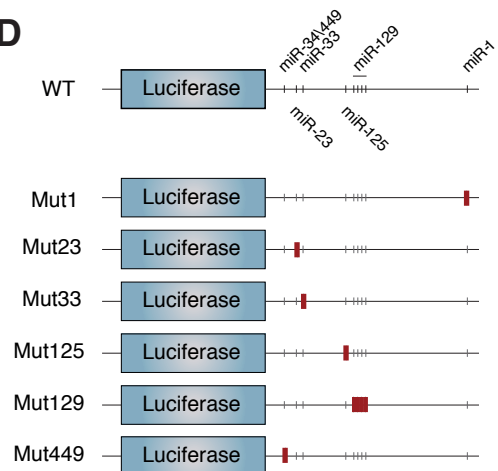

Supplement: Supplementary file 1 — Fig. S1. (A) Sequence alignment of the indicated miRs on human ZNF281 3’UTR. (B) Schematic representation of the human ZNF281 3’UTR and murine Zfp281 3’UTR indicating the binding sites of differentiation‐related miRs selected for further analysis. (C) Murine NIH3T3 cells were transfected with the indicated miRs and collected at different time points. WB analysis demonstrates that Zfp281 is not under control of miR‐23a/b; β‐actin was used as a loading control. The asterisk indicates non‐specific band. (D) Schematic representation of ZNF281 3’UTR different mutants. Red boxes indicate deletions of the relative binding sites along ZNF281 3’UTR. [file MOL2-14-294-s001.pdf]

## Supplementary Figure S2 related to Figure 2

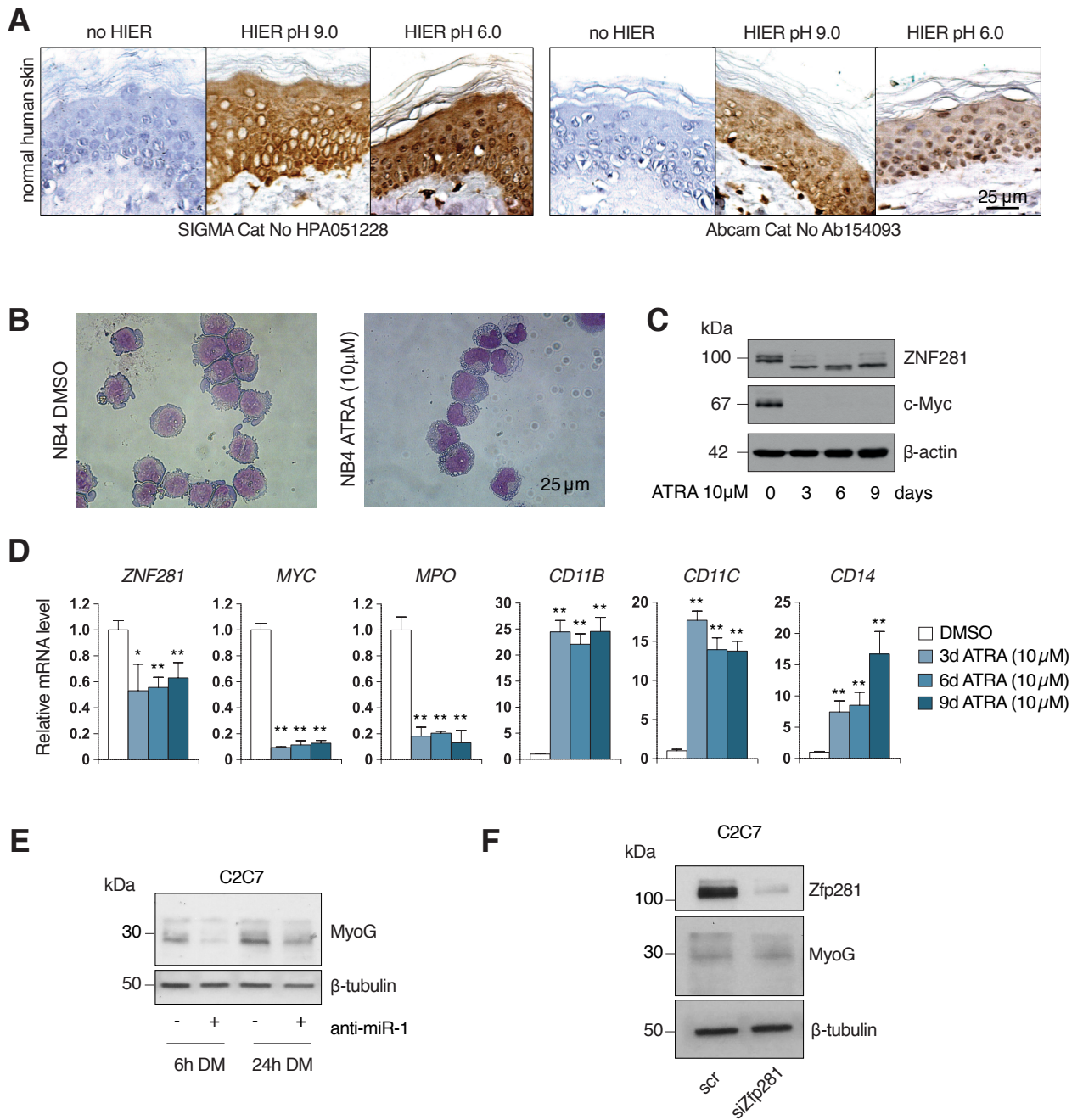

Supplement: Supplementary file 2 — Fig. S2. (A) Immunostaining of ZNF281 on normal human skin. Two different antibodies and three different conditions of heat‐induced epitope retrieval (no HIER, HIER pH9 EDTA, and HIER pH6 Citrate) were used to determine the optimal condition for immunostaining. (B) Representative images of NB4 cells treated with DMSO or ATRA 10 µM for 9 days. (C) WB analysis of NB4 cells induced to differentiate for the indicated times; ‐actin was used as a loading control. (D) qPCR analysis of samples in (C). (E) WB analysis of C2C7 cells treated with the indicated RNA oligonucleotides for 24 h and then shifted in differentiation medium for either 6 or 24 h; ‐tubulin was used as a loading control. (F) WB analysis of C2C7 cells transfected with the indicated siRNAs for 48h; ‐tubulin was used as a loading control. [file MOL2-14-294-s002.pdf]

Supplementary Figure S3 related to Figure 4

A

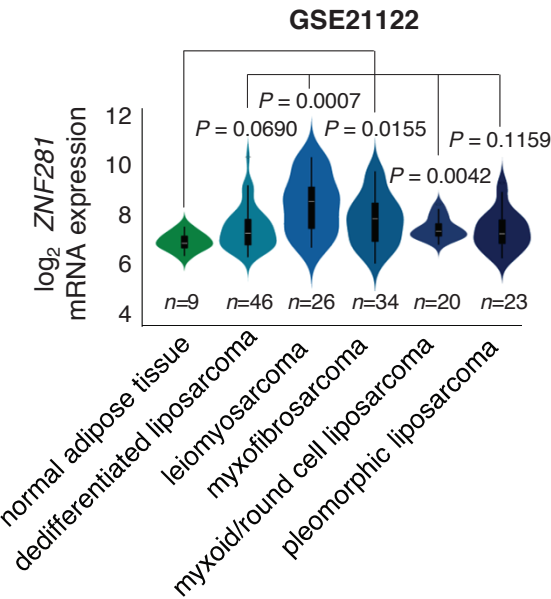

B

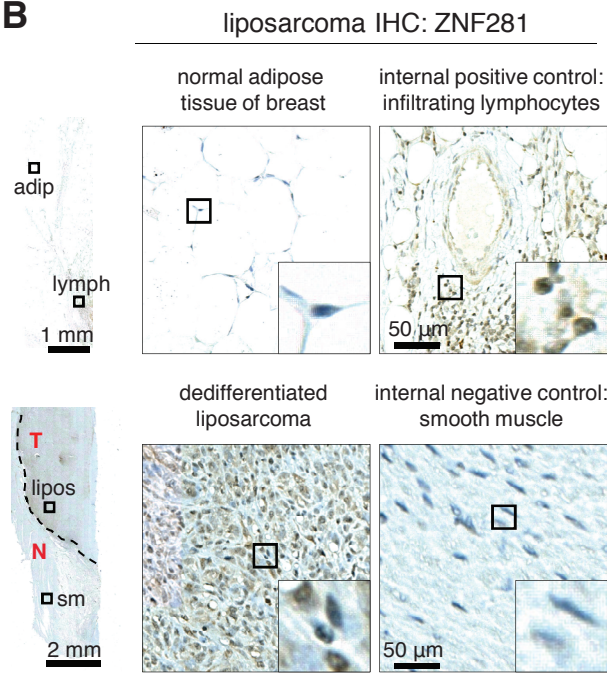

C

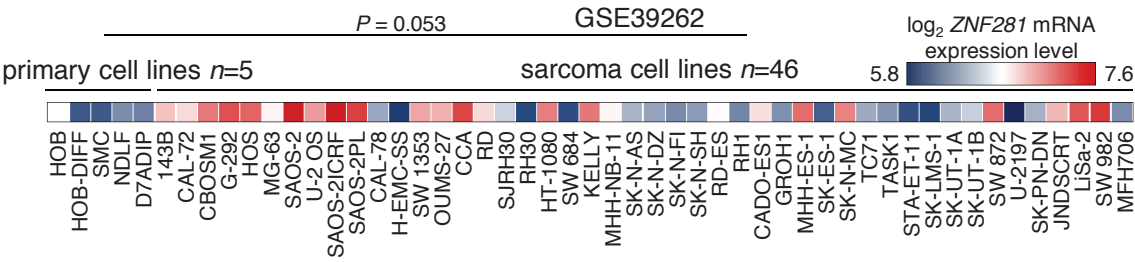

Supplement: Supplementary file 3 — Fig. S3. (A) Violin plots comparing ZNF281 expression between normal adipose tissue and different types of liposarcoma from the study GSE21122. (B) Immunostaining of ZNF281 on either normal human adipose tissue of breast or de‐differentiated liposarcoma. Infiltrating lymphocytes were used as internal positive control for ZNF281 immunostaining of breast, meanwhile smooth muscle adjacent to tumor was used as internal negative control for specificity of ZNF281 immunostaining of liposarcoma. (C) A heatmap showing the relative mRNA expression of ZNF281 in either 5 normal cell lines of soft tissues or 46 cell lines of soft tissue cancer. [file MOL2-14-294-s003.pdf]

# Supplementary Figure S4 uncropped western blots

WB related to Figure 1D

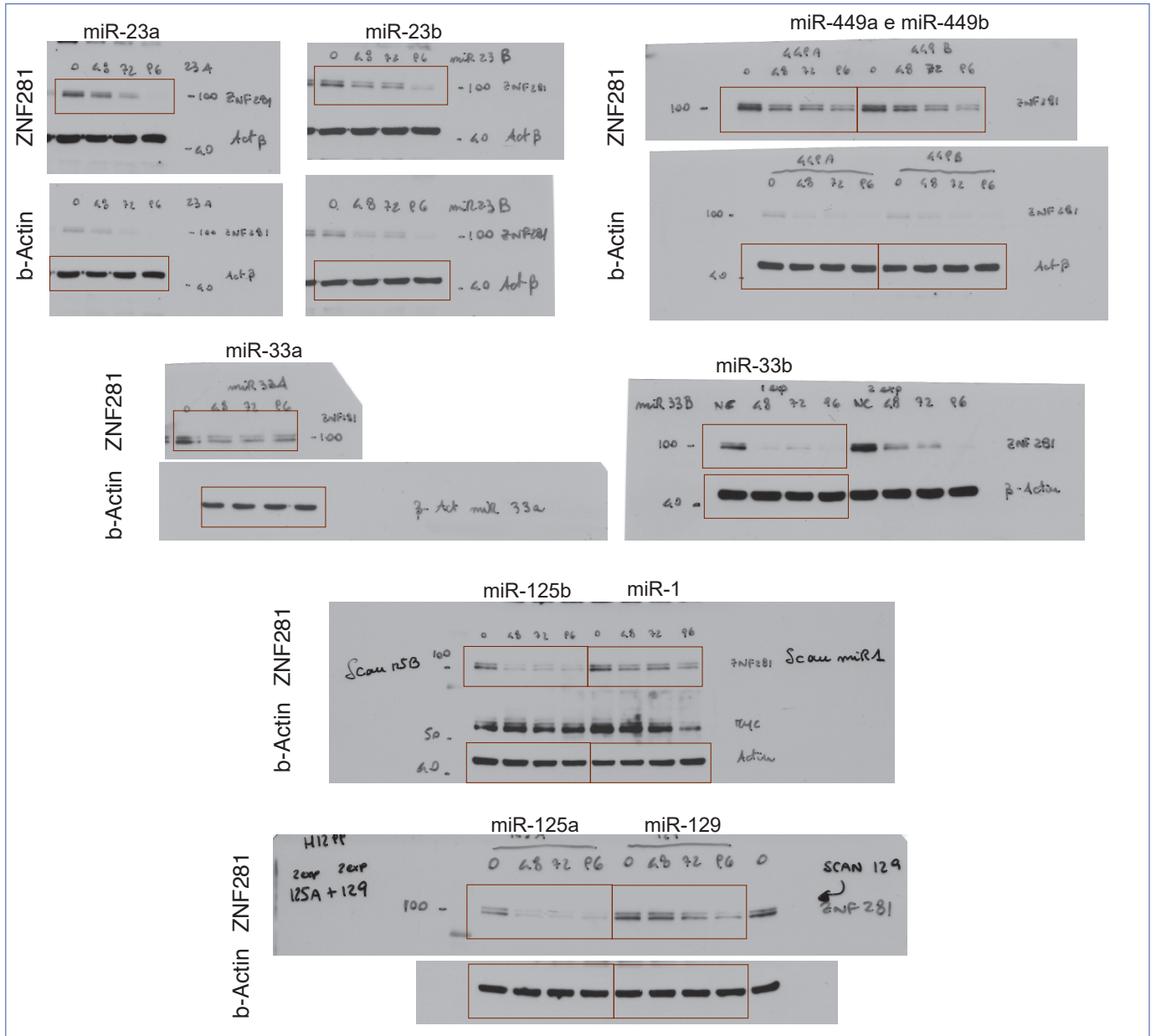

WB related to Figure 2B

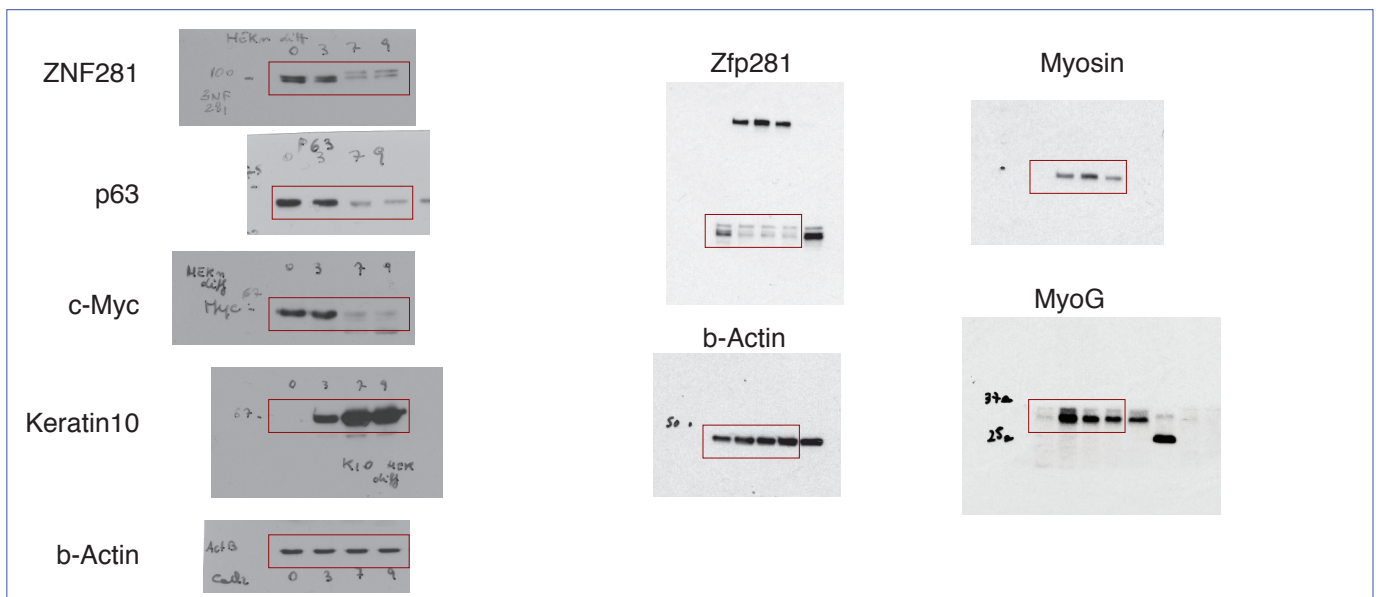

Supplement: Supplementary file 4 — Fig. S4. Uncropped western blots related to Figs 1D and 2B,C. [file MOL2-14-294-s004.pdf]
