# Supplementary material for: ZNF281/Zfp281 is a target of miR‐1 and counteracts muscle differentiation
Source: Mol Oncol. 2019 Dec 24;14(2):294–308. doi: 10.1002/1878-0261.12605 (PMC6998661; doi:10.1002/1878-0261.12605)

Supplementary Figure S5 uncropped western blots

WB related to Figure 2F

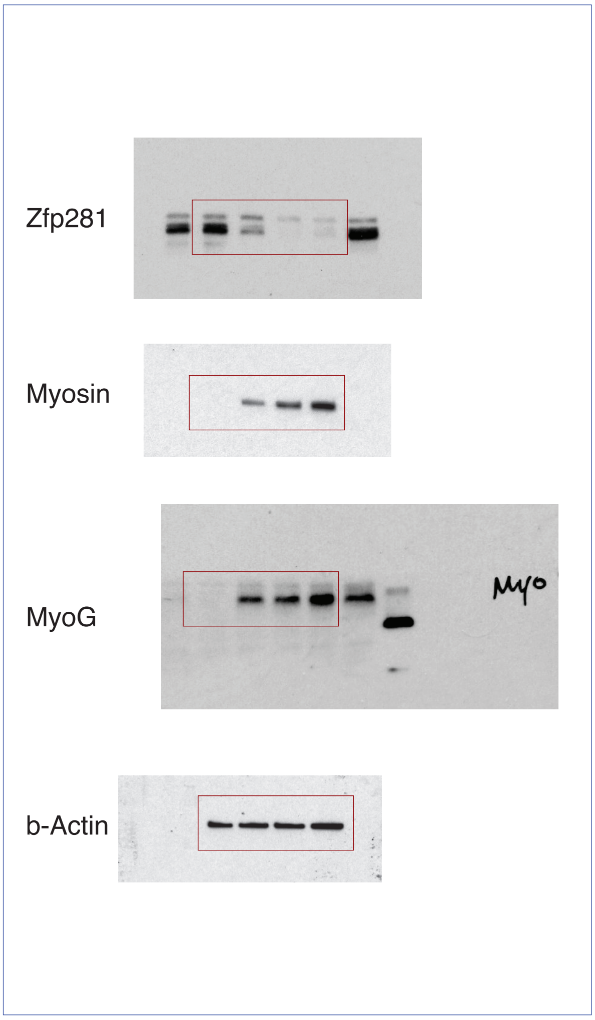

WB related to Figure 2G

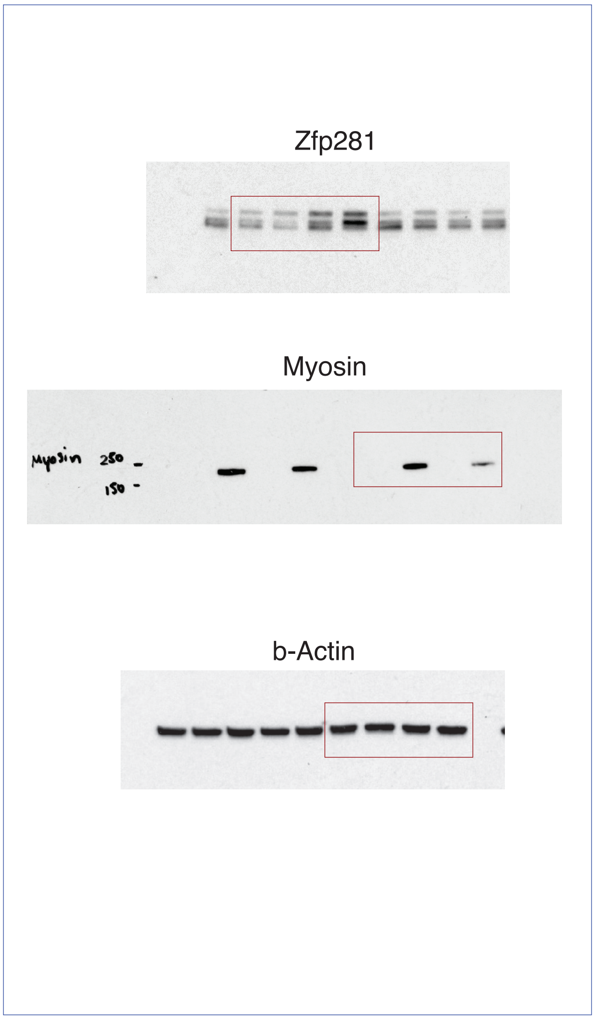

WB related to Figure S2C

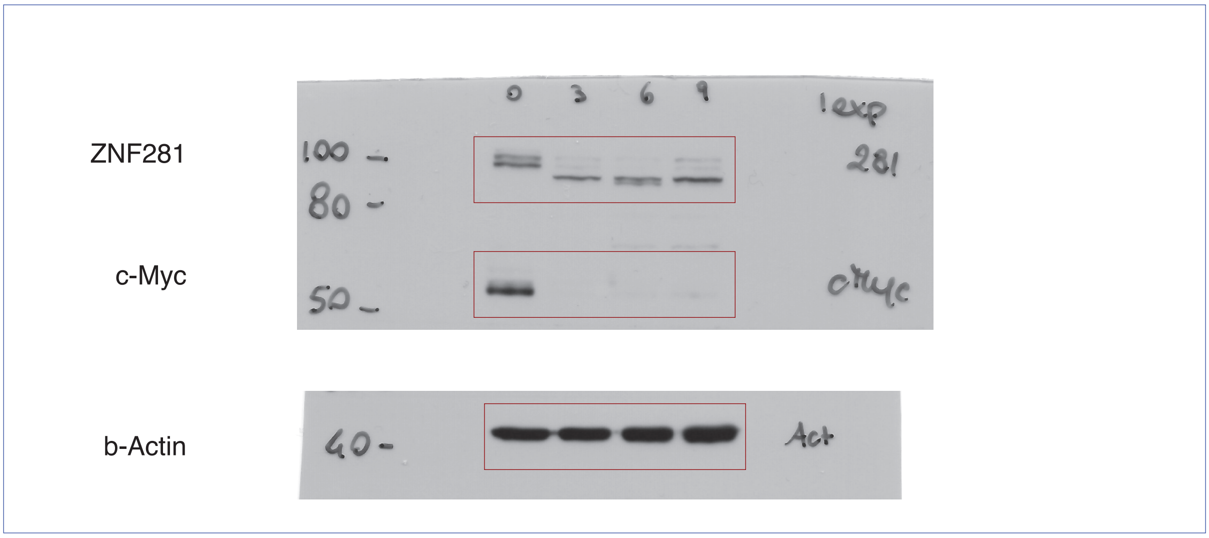

Supplement: Supplementary file 5 — Fig. S5. Uncropped western blots related to Fig. 2F,G and to Fig. S2C. [file MOL2-14-294-s005.pdf]
