# Supplementary material for: ZNF281/Zfp281 is a target of miR‐1 and counteracts muscle differentiation
Source: Mol Oncol. 2019 Dec 24;14(2):294–308. doi: 10.1002/1878-0261.12605 (PMC6998661; doi:10.1002/1878-0261.12605)
Supplement: Supplementary file 6 — Table S1 . Bioinformatic analysis of miRNA sites in human ZNF281 3'‐UTR. [file MOL2-14-294-s006.pdf]

Nicolai et al.
Supplementary Table S1.
Bioinformatic analysis of miRNA sites in human *ZNF281* 3'-UTR

MirTarget - miRDB 2019	Targetscan 7.1 (ver 2016)	miRanda (2010)
method: Support Vector Machine	method: Seed complementary	method: Complementary
http://mirdb.org	http://www.targetscan.org	http://www.microrna.org
**203 miRNAs**	**119 miRNAs**	**69 miRNAs**
**hsa-miR-1**	**hsa-miR-1**	**hsa-miR-1**
**hsa-miR-125a**	**hsa-miR-125a**	**hsa-miR-125a**
**hsa-miR-125b**	**hsa-miR-125b**	**hsa-miR-125b**
**hsa-miR-129**	**hsa-miR-129**	**hsa-miR-129**
**hsa-miR-196a**	**hsa-miR-196a**	**hsa-miR-196a**
**hsa-miR-196b**	**hsa-miR-196b**	**hsa-miR-196b**
**hsa-miR-203**	**hsa-miR-203**	**hsa-miR-203**
**hsa-miR-206**	**hsa-miR-206**	**hsa-miR-206**
**hsa-miR-23a**	**hsa-miR-23a**	**hsa-miR-23a**
**hsa-miR-23b**	**hsa-miR-23b**	**hsa-miR-23b**
**hsa-miR-320a**	**hsa-miR-320a**	**hsa-miR-320a**
**hsa-miR-320b**	**hsa-miR-320b**	**hsa-miR-320b**
**hsa-miR-320c**	**hsa-miR-320c**	**hsa-miR-320c**
**hsa-miR-320d**	**hsa-miR-320d**	**hsa-miR-320d**
**hsa-miR-33a**	**hsa-miR-33a**	**hsa-miR-33a**
**hsa-miR-33b**	**hsa-miR-33b**	**hsa-miR-33b**
**hsa-miR-34a**	**hsa-miR-34a**	**hsa-miR-34a**
**hsa-miR-34c**	**hsa-miR-34c**	**hsa-miR-34c**
**hsa-miR-382**	**hsa-miR-382**	**hsa-miR-382**
**hsa-miR-449a**	**hsa-miR-449a**	**hsa-miR-449a**
**hsa-miR-449b**	**hsa-miR-449b**	**hsa-miR-449b**
**hsa-miR-450a**	**hsa-miR-450a**	**hsa-miR-450a**
**hsa-miR-488**	**hsa-miR-488**	**hsa-miR-488**
**hsa-miR-494**	**hsa-miR-494**	**hsa-miR-494**
**hsa-miR-495**	**hsa-miR-495**	**hsa-miR-495**
**hsa-miR-613**	**hsa-miR-613**	**hsa-miR-613**
hsa-miR-10399	hsa-miR-101	hsa-let-7b
hsa-miR-10400	hsa-miR-122	hsa-let-7c
hsa-miR-106a	hsa-miR-124	hsa-let-7i
hsa-miR-1200	hsa-miR-136	hsa-miR-106a
hsa-miR-12127	hsa-miR-137	hsa-miR-106b
hsa-miR-12136	hsa-miR-139	hsa-miR-128
hsa-miR-124	hsa-miR-140	hsa-miR-137
hsa-miR-1248	hsa-miR-141	hsa-miR-17
hsa-miR-1272	hsa-miR-142	hsa-miR-181a
hsa-miR-1284	hsa-miR-145	hsa-miR-181b
hsa-miR-1321	hsa-miR-146	hsa-miR-181c
hsa-miR-141	hsa-miR-153	hsa-miR-181d
hsa-miR-152	hsa-miR-154	hsa-miR-183

hsa-miR-154	hsa-miR-186	hsa-miR-186
hsa-miR-1587	hsa-miR-188	hsa-miR-199a
hsa-miR-182	hsa-miR-19	hsa-miR-199b
hsa-miR-188	hsa-miR-194	hsa-miR-20a
hsa-miR-200a	hsa-miR-199	hsa-miR-20b
hsa-miR-2115	hsa-miR-200a	hsa-miR-216a
hsa-miR-2117	hsa-miR-200b	hsa-miR-219
hsa-miR-222	hsa-miR-200c	hsa-miR-22
hsa-miR-2278	hsa-miR-204	hsa-miR-223
hsa-miR-23c	hsa-miR-205	hsa-miR-300
hsa-miR-3065	hsa-miR-211	hsa-miR-30a
hsa-miR-3074	hsa-miR-212	hsa-miR-30b
hsa-miR-3124	hsa-miR-216a	hsa-miR-30c
hsa-miR-3129	hsa-miR-216b	hsa-miR-30d
hsa-miR-3133	hsa-miR-22	hsa-miR-30e
hsa-miR-3140	hsa-miR-223	hsa-miR-367
hsa-miR-3148	hsa-miR-224	hsa-miR-371
hsa-miR-3163	hsa-miR-23c	hsa-miR-374a
hsa-miR-3201	hsa-miR-24	hsa-miR-374b
hsa-miR-323a	hsa-miR-28	hsa-miR-375
hsa-miR-329	hsa-miR-302	hsa-miR-376c
hsa-miR-338	hsa-miR-31	hsa-miR-381
hsa-miR-3606	hsa-miR-320	hsa-miR-454
hsa-miR-362	hsa-miR-323	hsa-miR-485
hsa-miR-3620	hsa-miR-326	hsa-miR-543
hsa-miR-3652	hsa-miR-329	hsa-miR-7
hsa-miR-3658	hsa-miR-330	hsa-miR-708
hsa-miR-3664	hsa-miR-335	hsa-miR-875
hsa-miR-3686	hsa-miR-340	hsa-miR-9
hsa-miR-369	hsa-miR-34b	hsa-miR-98
hsa-miR-3714	hsa-miR-362	
hsa-miR-371a	hsa-miR-369	
hsa-miR-371b	hsa-miR-371	
hsa-miR-372	hsa-miR-371a	
hsa-miR-373	hsa-miR-371b	
hsa-miR-374a	hsa-miR-372	
hsa-miR-374b	hsa-miR-373	
hsa-miR-374c	hsa-miR-374	
hsa-miR-378a	hsa-miR-374c	
hsa-miR-3910	hsa-miR-375	
hsa-miR-3927	hsa-miR-376	
hsa-miR-3929	hsa-miR-381	
hsa-miR-3938	hsa-miR-384	
hsa-miR-3941	hsa-miR-409	
hsa-miR-3973	hsa-miR-410	
hsa-miR-4255	hsa-miR-411	
hsa-miR-4273	hsa-miR-421	
hsa-miR-4310	hsa-miR-429	
hsa-miR-4319	hsa-miR-431	
hsa-miR-4324	hsa-miR-4319	
hsa-miR-4328	hsa-miR-4429	
hsa-miR-4330	hsa-miR-450	

hsa-miR-4422	hsa-miR-451
hsa-miR-4429	hsa-miR-451a
hsa-miR-4430	hsa-miR-452
hsa-miR-4432	hsa-miR-485
hsa-miR-4478	hsa-miR-486
hsa-miR-4495	hsa-miR-487
hsa-miR-4499	hsa-miR-487a
hsa-miR-4517	hsa-miR-493
hsa-miR-451b	hsa-miR-499a
hsa-miR-4534	hsa-miR-501
hsa-miR-4639	hsa-miR-502
hsa-miR-466	hsa-miR-505
hsa-miR-4661	hsa-miR-506
hsa-miR-4666a	hsa-miR-520
hsa-miR-4672	hsa-miR-543
hsa-miR-4674	hsa-miR-5688
hsa-miR-4690	hsa-miR-582
hsa-miR-4732	hsa-miR-653
hsa-miR-4739	hsa-miR-655
hsa-miR-4756	hsa-miR-665
hsa-miR-4761	hsa-miR-668
hsa-miR-4772	hsa-miR-670
hsa-miR-4791	hsa-miR-6866
hsa-miR-4802	hsa-miR-7
hsa-miR-487a	hsa-miR-802
hsa-miR-493	hsa-miR-873
hsa-miR-499a	hsa-miR-876
hsa-miR-5003	hsa-miR-892
hsa-miR-506	
hsa-miR-512	
hsa-miR-513a	
hsa-miR-513b	
hsa-miR-513c	
hsa-miR-514b	
hsa-miR-518a	
hsa-miR-518b	
hsa-miR-518c	
hsa-miR-518d	
hsa-miR-518f	
hsa-miR-5197	
hsa-miR-526a	
hsa-miR-544b	
hsa-miR-548a	
hsa-miR-548ar	
hsa-miR-548at	
hsa-miR-548au	
hsa-miR-548av	
hsa-miR-548az	
hsa-miR-548bc	
hsa-miR-548c	
hsa-miR-548e	
hsa-miR-548f	

hsa-miR-548g
hsa-miR-548k
hsa-miR-548n
hsa-miR-5580
hsa-miR-5582
hsa-miR-5583
hsa-miR-568
hsa-miR-5681a
hsa-miR-5685
hsa-miR-5688
hsa-miR-5692a
hsa-miR-5692b
hsa-miR-5692c
hsa-miR-5699
hsa-miR-5700
hsa-miR-582
hsa-miR-603
hsa-miR-6079
hsa-miR-610
hsa-miR-6124
hsa-miR-6128
hsa-miR-616
hsa-miR-633
hsa-miR-649
hsa-miR-6499
hsa-miR-6504
hsa-miR-655
hsa-miR-670
hsa-miR-671
hsa-miR-6739
hsa-miR-6761
hsa-miR-6768
hsa-miR-6828
hsa-miR-6831
hsa-miR-6835
hsa-miR-6854
hsa-miR-6856
hsa-miR-6857
hsa-miR-6866
hsa-miR-6868
hsa-miR-6873
hsa-miR-7-1
hsa-miR-7106
hsa-miR-7152
hsa-miR-7156
hsa-miR-7157
hsa-miR-7-2
hsa-miR-7844
hsa-miR-7849
hsa-miR-8054
hsa-miR-8070
hsa-miR-8082

hsa-miR-8087
hsa-miR-8485
hsa-miR-885
hsa-miR-889
hsa-miR-9
hsa-miR-936
hsa-miR-95
hsa-miR-9983
