# Supplementary material for: ZNF281/Zfp281 is a target of miR‐1 and counteracts muscle differentiation
Source: Mol Oncol. 2019 Dec 24;14(2):294–308. doi: 10.1002/1878-0261.12605 (PMC6998661; doi:10.1002/1878-0261.12605)
Supplement: Supplementary file 9 — Table S4 . Tissue microarray TMA‐SO751a from US Biomax (leiomyosarcoma/rhabdomyosarcoma duplicate cores per case). [file MOL2-14-294-s009.pdf]

Nicolai et al.

Supplementary Table S4.

Tissue microarray TMA-SO751a from US Biomax (leiomyosarcoma/rhabdomyosarcoma duplicate cores per case)

Scoring of nuclear ZNF281 expression level:

intensity (0/1/2/3)
% pos cells (0%-100%)
histological "H-score" (as intens%: 0-300)*

Pos.	No.	Age	Sex	Organ	Pathology diagnosis	TNM	Type	Tissue ID.	nuclear ZNF281					
									intens	%	H-score	H-index patient	Patient ID	
A1	1	85	M	Skin	High malignant leiomyosarcoma	T2aN0M0 G3	Malignant	Sfb040082	1	20	20	40	Sfb040082	0
A2	2	85	M	Skin	High malignant leiomyosarcoma	T2aN0M0 G3	Malignant	Sfb040082	1	60	60			
A3	3	42	M	Peritoneum	Moderate malignant leiomyosarcoma	T2bN0M0 G2	Malignant	Aac020130	1	50	50	50	Aac020130	
A4	4	42	M	Peritoneum	Moderate malignant leiomyosarcoma	T2bN0M0 G2	Malignant	Aac020130	1	50	50			
A5	5	56	F	Skin	Moderate malignant leiomyosarcoma	T2bN0M0 G2	Malignant	Sfb040134	1	90	90	90	Sfb040134	
A6	6	56	F	Skin	Moderate malignant leiomyosarcoma	T2bN0M0 G2	Malignant	Sfb040134	1	90	90			
A7	7	74	F	Skin	Low malignant leiomyosarcoma	T1aN0M0 G1	Malignant	Sfb040277	0	0	0	0	Sfb040277	
A8	8	74	F	Skin	Low malignant leiomyosarcoma	T1aN0M0 G1	Malignant	Sfb040277	0	0	0			
A9	9	34	M	Skin	Moderate malignant leiomyosarcoma	T1bN0M0 G2	Malignant	Sfb040309	1	70	70	55	Sfb040309	
B1	10	34	M	Skin	Moderate malignant leiomyosarcoma	T1bN0M0 G2	Malignant	Sfb040309	1	40	40			
B2	11	20	F	Skin	Moderate malignant leiomyosarcoma	T1N0M0 G3	Malignant	Ssm020249	0	0	0	0	Ssm020249	
B3	12	20	F	Skin	Moderate malignant leiomyosarcoma	T1N0M0 G3	Malignant	Ssm020249	0	0	0			
B4	13	49	F	Skin	Moderate malignant leiomyosarcoma	T2aN0M0 G2	Malignant	Sft040268	1	40	40	35	Sft040268	
B5	14	49	F	Skin	Moderate malignant leiomyosarcoma	T2aN0M0 G2	Malignant	Sft040268	1	30	30			
B6	15	34	F	Skin	Moderate malignant epithelioid leiomyosarcoma	T2N0M0 G2	Malignant	Sft040287	1	60	60	60	Sft040287	
B7	16	34	F	Skin	Moderate malignant epithelioid leiomyosarcoma	T2N0M0 G2	Malignant	Sft040287	1	60	60			
B8	17	38	M	Skin	Low malignant leiomyosarcoma	T1aN0M0 G1	Malignant	Ssm030032	0,5	20	10	10	Ssm030032	
B9	18	38	M	Skin	Low malignant leiomyosarcoma	T1aN0M0 G1	Malignant	Ssm030032	0,5	20	10			
C1	19	44	F	Ligament	Low malignant leiomyosarcoma	T2bN0M0 G1	Malignant	Slm040092	0	0	0	0	Slm040092	
C2	20	44	F	Ligament	Low malignant leiomyosarcoma	T2bN0M0 G1	Malignant	Slm040092	0	0	0			
C3	21	69	F	bdominal cav	Moderate malignant leiomyosarcoma	T2bN0M0 G2	Malignant	Ssm020181	0	0	0	0	Ssm020181	
C4	22	69	F	bdominal cav	Moderate malignant leiomyosarcoma	T2bN0M0 G2	Malignant	Ssm020181	0	0	0			
C5	23	88	F	Skin	Low malignant leiomyosarcoma	T1aN0M0 G1	Malignant	Ssm010139	1	50	50	55	Ssm010139	
C6	24	88	F	Skin	Low malignant leiomyosarcoma	T1aN0M0 G1	Malignant	Ssm010139	1	60	60			
C7	25	54	F	Peritoneum	Malignant pleomorphic leiomyosarcoma	T2bN0M0 G3	Malignant	Ssm030062	1	80	80	80	Ssm030062	
C8	26	54	F	Peritoneum	Malignant pleomorphic leiomyosarcoma	T2bN0M0 G3	Malignant	Ssm030062	1	80	80			
C9	27	58	F	Skin	Low malignant leiomyosarcoma	T2aN0MO G2	Malignant	Ssm030036	2	30	60	90	Ssm030036	
D1	28	58	F	Skin	Low malignant leiomyosarcoma	T2aN0MO G2	Malignant	Ssm030036	2	60	120			
D2	29	57	F	Skin	Low malignant leiomyosarcoma	T2aN0M0 G1	Malignant	Ssm030079	2	60	120	187,5	Ssm030079	
D3	30	57	F	Skin	Low malignant leiomyosarcoma	T2aN0M0 G1	Malignant	Ssm030079	3	85	255			
D4	31	78	M	Chest wall	Moderate malignant leiomyosarcoma	T1aN0M0 G2	Malignant	Ssm020205	0	0	0	0	Ssm020205	
D5	32	78	M	Chest wall	Moderate malignant leiomyosarcoma	T1aN0M0 G2	Malignant	Ssm020205	0	0	0			
D6	33	52	F	Perineum	Moderate malignant leiomyosarcoma	T1aN0M0 G2	Malignant	Ssm030067	0	0	0	0	Ssm030067	
D7	34	52	F	Perineum	Moderate malignant leiomyosarcoma	T1aN0M0 G2	Malignant	Ssm030067	0	0	0			
D8	35	31	M	Mesentery	Moderate malignant leiomyosarcoma	T1aN0M0 G2	Malignant	Amt020126	0	0	0	0	Amt020126	
D9	36	31	M	Mesentery	Moderate malignant leiomyosarcoma (fibrous tissu	T1aN0M0 G2	Malignant	Amt020126	0	0	0			
E1	37	16	M	Testis	Pleomorphic rhabdomyosarcoma	T1bN0M0 G2	Malignant	Mtt040096	0	0	0	0	Mtt040096	
E2	38	16	M	Testis	Pleomorphic rhabdomyosarcoma	T1bN0M0 G2	Malignant	Mtt040096	0	0	0			
E3	39	40	M	etroperitoneu	Embryonic rhabdomyosarcoma	T1bN0M0 G3	Malignant	Sst040330	0	0	0	0	Sst040330	
E4	40	40	M	etroperitoneu	Embryonic rhabdomyosarcoma	T1bN0M0 G3	Malignant	Sst040330	0	0	0			
E5	41	30	M	Skin	Pleomorphic rhabdomyosarcoma	T2aN0M0 G2	Malignant	Srm030089	1	70	70	70	Srm030089	
E6	42	30	M	Skin	Pleomorphic rhabdomyosarcoma	T2aN0M0 G2	Malignant	Srm030089	1	70	70			
E7	43	67	F	Cervix	Spindle cell rhabdomyosarcoma	T2bN0M0 G3	Malignant	Fdu040953	0	0	0	0	Fdu040953	
E8	44	67	F	Cervix	Spindle cell rhabdomyosarcoma	T2bN0M0 G3	Malignant	Fdu040953	0	0	0			
E9	45	50	M	Skin	Pleomorphic rhabdomyosarcoma	T1aN0M0 G2	Malignant	Srm040370	2	70	140	130	Srm040370	
F1	46	50	M	Skin	Pleomorphic rhabdomyosarcoma	T1aN0M0 G2	Malignant	Srm040370	2	60	120			
F2	47	91	M	Skin	Rhabdomyosarcoma	T2aN0M0 G3	Malignant	Srm030172	0	0	0	0	Srm030172	
F3	48	91	M	Skin	Rhabdomyosarcoma	T2aN0M0 G3	Malignant	Srm030172	0	0	0			
F4	49	33	F	keletal muscl	Embryonic rhabdomyosarcoma	T1aN0M0 G2	Malignant	Sfb040332	0	0	0	0	Sfb040332	
F5	50	33	F	keletal muscl	Embryonic rhabdomyosarcoma	T1aN0M0 G2	Malignant	Sfb040332	0	0	0			
F6	51	51	F	Cervix	Pleomorphic rhabdomyosarcoma	T2bN0M0 G3	Malignant	Fdu041147	0	0	0	0	Fdu041147	
F7	52	51	F	Cervix	Pleomorphic rhabdomyosarcoma	T2bN0M0 G3	Malignant	Fdu041147	0	0	0			
F8	53	74	F	Bladder	Pleomorphic rhabdomyosarcoma	T2bN0M0 G3	Malignant	Ubd040458	0	0	0	0	Ubd040458	
F9	54	74	F	Bladder	Pleomorphic rhabdomyosarcoma	T2bN0M0 G3	Malignant	Ubd040458	0	0	0			
G1	55	32	F	Cervix	Pleomorphic rhabdomyosarcoma	T2bN0M0 G3	Malignant	Fdu041182	0	0	0	0	Fdu041182	
G2	56	32	F	Cervix	Pleomorphic rhabdomyosarcoma	T2bN0M0 G3	Malignant	Fdu041182	0	0	0			
G3	57	49	F	Uterine cervix	Embryonic rhabdomyosarcoma	T2bN0M0 G3	Malignant	Fur041163	0	0	0	0	Fur041163	
G4	58	49	F	Uterine cervix	Embryonic rhabdomyosarcoma	T2bN0M0 G3	Malignant	Fur041163	0	0	0			
G5	59	49	M	Testis	Embryonic rhabdomyosarcoma	T2bN0M0 G2	Malignant	Mtt030229	2	90	180	180	Mtt030229	
G6	60	49	M	Testis	Embryonic rhabdomyosarcoma	T2bN0M0 G2	Malignant	Mtt030229	2	90	180			
G7	61	21	M	Peritoneum	Pleomorphic rhabdomyosarcoma	T1bN0M0 G3	Malignant	Apo040019	2	70	140	160	Apo040019	
G8	62	21	M	Peritoneum	Pleomorphic rhabdomyosarcoma	T1bN0M0 G3	Malignant	Apo040019	2	90	180			
G9	63	18	M	Oral cavity	Alveolus rhabdomyosarcoma	T1bN0M0 G3	Malignant	Doc030135	1	50	50	50	Doc030135	
H1	64	18	M	Oral cavity	Alveolus rhabdomyosarcoma	T1bN0M0 G3	Malignant	Doc030135	1	50	50			
H2	65	40	F	Peritoneum	Pleomorphic rhabdomyosarcoma	T2bN0M0 G3	Malignant	Aac040131	1	50	50	50	Aac040131	
H3	66	40	F	Peritoneum	Pleomorphic rhabdomyosarcoma	T2bN0M0 G3	Malignant	Aac040131	1	50	50			
H4	67	23	F	Cavitas pelvis	Alveolus rhabdomyosarcoma	T2bN0M0 G3	Malignant	Apc040155	1	50	50	50	Apc040155	
H5	68	23	F	Cavitas pelvis	Alveolus rhabdomyosarcoma	T2bN0M0 G3	Malignant	Apc040155	1	50	50			
H6	69	48	M	Aynovium	Spindle cell rhabdomyosarcoma	T2N0M0 G2	Malignant	Sst040026	0	0	0	0	Sst040026	
H7	70	48	M	Aynovium	Spindle cell rhabdomyosarcoma	T2N0M0 G2	Malignant	Sst040026	0	0	0			
H8	71	10	M	keletal muscl	Embryonic rhabdomyosarcoma	T2aN0M0 G2	Malignant	Srm040374	3	90	270	205	Srm040374	
H9	72	10	M	keletal muscl	Embryonic rhabdomyosarcoma	T2aN0M0 G2	Malignant	Srm040374	2	70	140			
I1	73	56	F	Uterine cervix	Smooth muscle tissue of Uterine cervix	-	NAT	Aac140068	0	0	0	0	Aac140068	
I2	74	8	F	keletal muscl	Skeletal muscle tissue	-	Normal	Ssm04N012	0	0	0	0	Ssm04N012	
I3	75	26	M	Heart	Cardiac muscle tissue	-	Normal	Cht03N014	0	0	0	0	Cht03N014	
