# Supplementary material for: ZNF281/Zfp281 is a target of miR‐1 and counteracts muscle differentiation
Source: Mol Oncol. 2019 Dec 24;14(2):294–308. doi: 10.1002/1878-0261.12605 (PMC6998661; doi:10.1002/1878-0261.12605)
Supplement: Supplementary file 10 [file MOL2-14-294-s010.docx]

**Supplementary Figure Legends**

**Supplementary Figure S1**

(A) Sequence alignment of the indicated miRs on human ZNF281 3’UTR. (B) Schematic representation of the human ZNF281 3’UTR and murine Zfp281 3’UTR indicating the binding sites of differentiation-related miRs selected for further analysis. (C) Murine NIH3T3 cells were transfected with the indicated miRs and collected at different time points. WB analysis demonstrates that Zfp281 is not under control of miR-23a/b; β-actin was used as a loading control. The asterisk indicates non-specific band. (D) Schematic representation of ZNF281 3’UTR different mutants. Red boxes indicate deletions of the relative binding sites along ZNF281 3’UTR.

**Supplementary Figure S2**

(A) Immunostaining of ZNF281 on normal human skin. Two different antibodies and three different conditions of heat-induced epitope retrieval (no HIER, HIER pH9 EDTA, and HIER pH6 Citrate) were used to determine the optimal condition for immunostaining.

(B) Representative images of NB4 cells treated with DMSO or ATRA 10 μM for 9 days.

(C) WB analysis of NB4 cells induced to differentiate for the indicated times; β-actin was used as a loading control.

(D) qPCR analysis of samples in (C).

(E) WB analysis of C2C7 cells treated with the indicated RNA oligonucleotides for 24 hours and then shifted in differentiation medium for either 6 or 24 hours; β-tubulin was used as a loading control.

(F) WB analysis of C2C7 cells transfected with the indicated siRNAs for 48h; β-tubulin was used as a loading control.

**Supplementary Figure S3**

(A) Violin plots comparing *ZNF281* expression between normal adipose tissue and different types of liposarcoma from the study GSE21122.

(B) Immunostaining of ZNF281 on either normal human adipose tissue of breast or de-differentiated liposarcoma. Infiltrating lymphocytes were used as internal positive control for ZNF281 immunostaining of breast, meanwhile smooth muscle adjacent to tumor was used as internal negative control for specificity of ZNF281 immunostaining of liposarcoma.

(C) A heatmap showing the relative mRNA expression of *ZNF281* in either 5 normal cell lines of soft tissues or 46 cell lines of soft tissue cancer.

**Supplementary Figure S4**

Uncropped western blots related to Fig. 1D and 2B and 2C

**Supplementary Figure S5**

Uncropped western blots related to Fig. 2F and 2G and to Supplementary Fig. S2C
